# Patient-Reported OUtcome measures in key African languages to promote Diversity in research and clinical practice (PROUD)—protocol for a systematic review of measurement properties

**DOI:** 10.1186/s13063-021-05328-z

**Published:** 2021-06-05

**Authors:** Martin Heine, Lidwine B. Mokkink, Chanel van Zyl, Wayne Derman, Susan Hanekom

**Affiliations:** 1 Institute of Sport and Exercise Medicine, Division of Orthopaedics, Department of Surgical Sciences, Faculty of Medicine and Health Sciences, Stellenbosch University, Francie van Zyl drive, Cape Town, 8000 South Africa; 2 Amsterdam UMC, Vrije Universiteit Amsterdam, Department of Epidemiology and Data Science, Amsterdam Public Health Research Institute, Amsterdam, The Netherlands; 3 Division of Physiotherapy, Department of Health and Rehabilitation Sciences, Faculty of Medicine and Health Sciences, Stellenbosch University, Cape Town, South Africa

**Keywords:** Patient-reported outcome measures, Sub-Saharan Africa, Review

## Abstract

**Introduction:**

Sub-Saharan Africa is a subcontinent with a proud cultural richness and diversity, yet inexplicably also a region with severe health care challenges and inequity. To challenge this health equity gap and reduce the burden of disease, the patient’s voice in monitoring and evaluation of health and health care interventions is paramount. The aim of this two-phased review is to map the availability of patient-reported outcome measures (PROMs) in a selection of non-English, African Languages, and systematically evaluate the measurement properties of the PROMs that were identified.

**Methods:**

This systematic review will be conducted in two phases. In phase 1, we will scope the literature for patient-reported outcome measures (PROMs), either developed from scratch or through translation and validation in a sub-Saharan African country and a selection of *non-English*, African languages (n = 31; spoken in > 10 million people and/or a national language). The availability of PROMs will be mapped against the previously reported burden of disease in the respective countries included. Subsequently, in phase 2, we systematically evaluate the measurement properties of these PROMs using the COnsensus-based Standards for the selection of health Measurement INstruments (COSMIN) methodology for systematic reviews on PROMs. To ensure rigour, secondary searches will be developed to specifically locate articles that report on the measurement properties of the PROMs identified during phase 1. The evidence will be graded using the modified GRADE approach.

**Discussion:**

This review will provide a comprehensive overview and quality appraisal of PROMs developed in non-English, African languages. Consequently, this review when concluded may be an important first step in promoting access to these PROMs for use in clinical practice and research, as well as facilitate identification and prioritization of key knowledge gaps.

**Supplementary Information:**

The online version contains supplementary material available at 10.1186/s13063-021-05328-z.

## Background

The World Health Organization (WHO) defines universal health coverage as “all people having access to the health services they need, when and where they need them, without financial hardship”. It includes the full range of essential health services, from health promotion to prevention, treatment, rehabilitation, and palliative care [1]. Furthermore, it is highlighted that good health care systems are rooted in communities and focus not only on preventing and treating disease and illness but also on helping to improve well-being and quality of life [1]. The latter, well-being and quality of life, are two prime examples of patient-reported outcomes; outcomes commonly assessed using patient-reported outcome measures (PROMs). Some examples of common PROMs for health-related quality of life include the EuroQol EQ-5D or the Short Form 36 Health Survey [2, 3]. Such PROMs are increasingly used to assess an aspect of a patient’s health status that can be directly derived from the patient without interpretation of the patient’s response by anyone other than the patient [4]. Well-being and quality of life are merely two examples, and other patient-reported outcomes are those related to mental health (e.g. depression, anxiety), health literacy (e.g. disease knowledge or attitude), measures of activity (e.g. physical activity), or societal participation amongst others. Albeit used increasingly, in particular with the context of clinical trials, there have been repeated calls for the inclusion of the “patient’s voice” in the real-world context during prospective data collection [5–7].

Sub-Saharan Africa is a world region characterised by a proud and rich diversity in cultures and languages. However, sub-Saharan Africa is also a world region with rapidly increasing levels of multidimensional poverty [8], and a shifting burden of disease (e.g. communicable towards non-communicable disease) [9], financial constraints, geographical challenges, and lack of human resources [10]. Amongst others, these pertinent factors perpetuate a complex system of health inequality. One can argue that the richness and diversity in languages and cultures (in sub-Saharan Africa), in combination with complex low-resource settings, complicates adequate and comprehensive evaluation in a clinical and/or academic context. Innovative and bottom-up approaches are required in tackling these complex challenges (e.g. health inequality) [11], while safeguarding the tremendous richness and diversity. High-quality PROMs, suitable to the local context (e.g. language, cultural validity), may assist such bottom-up innovation by promoting inclusivity throughout academia and health care.

The average number of living languages per country in sub-Saharan Africa is estimated to be 57, while in some countries (e.g. Nigeria) as many as > 500 recognised languages are spoken [12]. In about half (46%) of the forty-eight sub-Saharan African countries, English is one of the commonly spoken languages [12]. Yet, despite English being a common language, only a mere ~ 16% of the total sub-Saharan African population speaks some level of English (> 169 million people); either as a first language or second language (see online supplement 1) [12]. Other common languages spoken include Swahili (> 108 million), Arabic (> 88 million), French (> 75 million), and Hausa (> 71 million). With most PROMs developed in languages commonly spoken in “developed” countries, one can argue that there may be a significant gap in the availability of (and access to) PROMs that are linguistically and contextually valid. In light of the shifting burden of disease [9, 13–15], such gaps may be more pronounced for some outcomes or some disease clusters [16]. The overarching aim of this review is therefore to improve access to contextually validated and language-appropriate PROMs of high quality, to improve their use in academia and clinical practice, and consequently to facilitate the inclusivity of more patients’ voices.

The objectives of this review are therefore threefold (i) to scope the literature of studies conducted in sub-Saharan Africa on the use and measurement properties of PROMs in commonly spoken sub-Saharan African languages; (ii) indirectly, map the availability of non-English PROMs against the most recent reported burden of disease and International Classification of Functioning (ICF); and (iii) systematically evaluate the measurement properties of the PROMs identified under the first objective.

## Methods/design

**Fig. 1:**
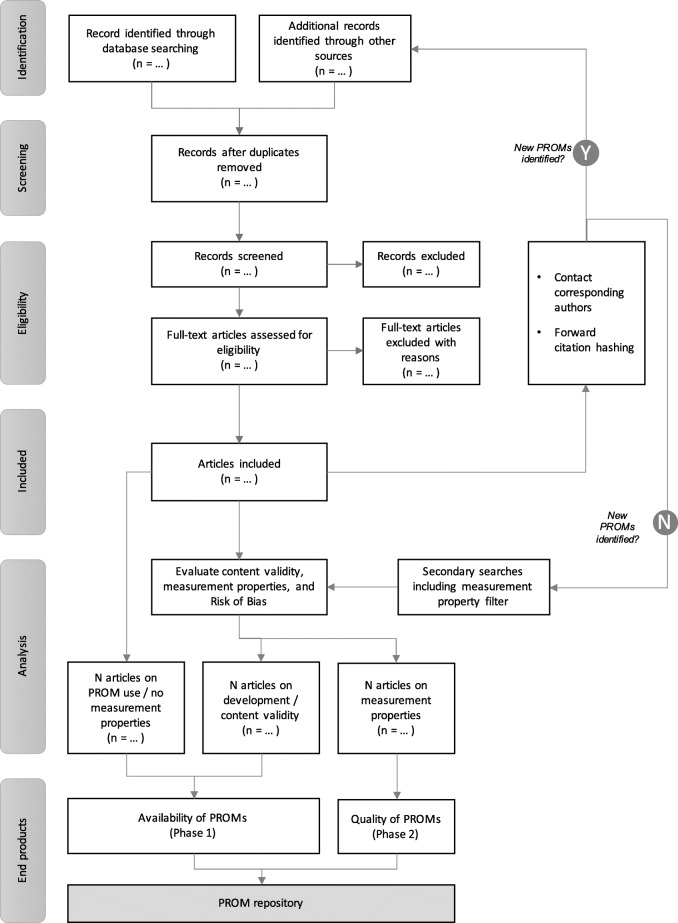
PRISMA-based flowchart. Articles that report on the use, development, or measurement properties of a PROM will be included. Corresponding authors will be contacted to consult on additional PROMs that may not have arisen from the search, and forward citation hashing will be used to identify articles that cited the work included and are deemed eligible for inclusion. Once this iterative process does not lead to new PROMs being identified phase 1 has been completed (availability). Moving to phase 2, secondary searches will be performed for each identified PROM to ensure all articles about measurement properties for each of the identified PROMs are allocated and considered. The quality of all individual studies reporting on measurement properties is evaluated using standard criteria. End products include a minimum of two systematic reviews (availability of PROMs, and on the quality of PROMs) as well as the start of a PROM repository

This systematic review will comprise two phases, based on a single yet broad selection of studies (see Fig. 1). The first phase entails scoping the literature to identify studies that report on (i) the development (e.g. translation), (ii) use and/or (iii) evaluation (e.g. validity, reliability) of PROMs in non-English languages commonly spoken (see Table 1) and conducted in one of 48 sub-Saharan African countries. Once a rigorous overview of PROMs has been established based on the broad and scoping search, in phase 2, we will systematically review the measurement properties for each of the PROMs identified by systematically collating the results from articles relevant to that specific PROM. The systematic evaluation of measurement properties will be conducted as guided by the procedures outlined by the Consensus-based standards for the Selection of Health Measurement Instruments (COSMIN) group [2, 11, 13, 14] and will be registered as a systematic review in an applicable repository at the time (e.g. PROSPERO).

**Table 1:** Overview of languages included relative to the total population. Level indicates the national (1), provincial (2), or used for wider communication (3). See online supplement 1 for the comprehensive overview. Languages spoken by > 10 million people plus national languages (level 1) are included

		Summary statistics		
	Total population	1,072,128,000		
	N English speaking	169,531,510		
	Mean (SD) living languages	57 (85)		
	Mean (SD) literacy rate	65% (20%)		
** Languages spoken by > 10 million**	**Language**	**N spoken**	**Level**	**Ranking (max 94)**
	**Swahili**	108,888,800	1	1
	**French**	88,135,710	1	2
	**Arabic**	75,160,000	1	3
	**Hausa**	71,219,000	2	4
	**Pidgin**	62078,000	3	5
	**Amharic**	56,900,000	1	6
	**Yoruba**	42,209,000	2	7
	**Portuguese**	29,311,250	1	8
	**Igbo**	29,000,000	2	9
	**Zulu**	27,300,000	1	10
	**Sotho**	27,230,000	1	11
	**Xhosa**	19,150,000	1	12
	**Oromo**	19,100,000	2	13
	**Afrikaans**	17,287,000	1	14
	**Fulfulde**	16,169,000	3	15
	**Somali**	14,635,600	1	16
	**Setswana**	13,630,000	1	17
	**Wolof**	12,522,000	3	18
	**Jula**	12,504,000	3	19
	**Kinyarwanda**	11,600,000	1	20
	**Rundi**	10,420,000	1	21
	**Ibibio**	10,380,000	3	22
**+ National languages**	**Malagasy**	7,520,000	1	28
	**Tsonga**	5,680,000	1	36
	**Sango**	5,100,000	1	38
	**Swati**	4,800,000	1	40
	**Ndebele**	4,100,000	1	42
	**Venda**	2,910,000	1	48
	**Creole**	2,151,200	1	52
	**Spanish**	787,000	1	67
	**Kabuverdianu**	492,000	1	76

### Identify relevant studies

A comprehensive and broad initial search strategy has been developed for the identification of relevant studies and PROMs in PubMed, Web of Science, CINAHL, and AfricaWide. An example of the search strategy (PubMed) can be found in online supplement 2. In short, the search combines the following:

(i) All countries in sub-Saharan Africa according to the World Bank (medical subject headings, and Title/Abstract),
(ii) the 31 languages (see Table 1) spoken by > 10 million people supplemented with national languages spoken by < 10 million people [12], and
(iii) a search block for the identification of PROMs (developed by Oxford University; available at www.cosmin.nl).

Due to the initial scoping nature of our inquiry, the framework provided by Arksey and O’Malley will be followed while cognisant of the refinements suggested by the Joanna Briggs Institute and others [17–19]. Amendments to the search strategy may be made as the research team gets more familiar with the body of evidence. Three methods have been built in, in addition to the comprehensive search, to support rigour. First, grey literature will be sought using the forward citation hashing in Google Scholar of articles included in phase 1. The addition of grey literature is deemed particularly important in the African context, where access to publishing in (open-access) international journals is often challenging [20]. Second, corresponding authors of included articles will be consulted to advise on any additional PROMs they are aware of, in any of the languages indicated, beyond those identified during the initial study selection process. This process is repeated until no further PROMs are being identified. If no new PROMs are identified, secondary searches will be conducted for each of the identified PROMs (including name variations, abbreviations, etc.) in conjunction with the country search block and a search block to identify any articles that evaluate measurement properties specifically that may have been missed during the broad search [21].

### Study selection

Studies will be screened against the following preliminary inclusion and exclusion criteria, however, in line with the scoping nature of this initial phase, these can be revised once the research team gains more familiarity with the body of evidence [18]. Initial inclusion criteria require that the study is conducted in one of the sub-Saharan African countries, as identified by the World Bank (see online supplement 1) and reports on one or more measurement properties, the use of a PROM in clinical research, the development of a PROM, or the evaluation of the interpretability of the PROMs of interest in line with the COSMIN framework [4, 22].

In the instance that studies explicitly report on the use of a PROM without reporting on one or more of the measurement properties, a concerted effort will be made to track back to the original work in developing that PROM. No language or time restrictions will be applied during the search, and a concerted effort will be made to obtain assistance in case articles surface in a non-English language that is outside the language skillset of the research team (i.e. Afrikaans, French). A first initial screening of study titles will be conducted by a single reviewer (MH). Subsequently, titles and abstracts are assessed for potentially relevant articles and the selection of abstracts for full-text review will be conducted by two independent reviewers (MH and CvZ). If a study seems relevant by at least one reviewer based on the abstract, or in case of doubt, the article will be pushed to full-text review. Inclusion and exclusion of full-text articles are done by two reviewers independently, and a third reviewer will be consulted in case of irreconcilable disagreements between the first two reviewers. The study selection process will be streamlined using the open-access platform CADIMA (www.cadima.info).

In this review, a PROM is defined as “any report of the status of a patient's health condition that comes directly from the patient, without interpretation of the patient's response by a clinician or other, and assessed using self-administered questionnaires” [23]. Structured questionnaires, though completed by an observer (so-called ObSROMs), are also eligible for inclusion. ObSROMs are those in which “observations can be made, appraised, and recorded by a person other than the patient (e.g. caregiver) and do not require specialized professional training” [24]. While these ObSROMs provide an appraisal from the viewpoint of an observer (rather than directly from the patient), we deem the inclusion of these ObSROMs particularly valuable in relation to some of the adverse social determinants of health (e.g. low literacy).

### Phase 1: scoping review on all available PROMs

The included articles will be evaluated in two distinct phases. In phase 1, a charting form will be developed using an iterative process (to allow for refinement early in the data extraction process), and include the following: first author, title, year, article type (e.g. journal article, thesis), journal or article source, country, PROM characteristics (name, version, outcome measured by the PROM, licensing model/accessibility), making use of the ICF where possible [25], language(s), original language of development (e.g. English), recall period, target population, number of subscales and items, mode of administration, time for completion), study populations (e.g. healthy, specific condition, or age group), and purpose of use of the PROM (i.e. use in a trial, development of PROM or evaluation of measurement properties assessed (e.g. content validity)). This phase provides us with an overview of all available PROMs in any of the included languages. The availability of PROMs will subsequently be mapped against the burden of disease in the respective countries included by using (i) the Institute for Health Metrics and Evaluation recurring global burden of disease study [13], (ii) the proportion of the population in each country that is proficient in the languages included [12], and (iii) the ICF model [25]. Mapping the availability of PROMs relative to the burden of disease, across the proportion of the population that is proficient in specific languages, and aligned with the ICF model will be a first step in identifying potential knowledge gaps. Albeit, additional strategies will be needed to inform evidence-based recommendations (see Dissemination and future perspectives).

Data extraction is conducted by one reviewer and verified by a second reviewer. The data extraction process will be piloted on a random sample of six articles with percentage agreement needing to be > 80% across reviewers to begin formal extraction [4, 26, 27]. Any disagreements will be discussed, the data-extraction template revised if applicable, and a new random selection of six articles is made until agreement is > 80%.

### Phase 2: a systematic review of the quality of PROMs

In phase 2, an overview of all evidence of the quality of all available PROMs will be obtained. All studies in which a (version of a) PROM was developed (e.g. translated or developed from scratch), or in which one or more of the measurement properties were evaluated will be included in this review. The COSMIN methodology for systematic reviews of PROMs will be used to conduct the review [4], and the review will be registered in a relevant repository. In summary, after extracting the data from each article (e.g. results about the measurement properties, interpretability, and feasibility aspects) the study quality will be assessed using the COSMIN checklist [26, 28], and the results per study are compared against the criteria of good measurement properties (see Table 1 in Prinsen et al. 2018) [4]. Per PROM, and measurement property, all evidence will be summarized, and the quality of the evidence graded, using a modified GRADE approach [4]. Measurement properties will be defined in line with the published taxonomy [22], and assessed where applicable, in the following order:

- PROM development, content validity (including face validity) [28]
- Structural validity, internal consistency, cross-cultural validity, measurement invariance
- Reliability, measurement error, criterion validity, hypotheses testing for construct validity, and responsiveness.

Theoretically, no judgement can be made on the quality of a PROM when information on the content validity of that PROM is lacking [28]. However, as it is not unlikely that a PROM has been developed in language A, and only additional measurement properties (e.g. cross-cultural validity) in language B, all measurement properties are considered; even in the absence of reports on content validity for a specific PROM in a sub-Saharan African language.

The quality assessment of each study, quality assessment of each PROM (i.e. applying the criteria for good measurement properties against each result), and the grading of the evidence is conducted by two independent reviewers. This phase provides us with an overview of all evidence for the quality (i.e. regarding the nine measurement properties) that is available for each PROM, available in any of the included languages, and will be reported according to set reporting guidelines [29]. It is not our immediate aim to develop recommendations about the most suitable PROM to use, as we will include PROMs for any outcome, and various patient populations; the aim is to collect the evidence derived from phases 1 and 2 into a PROM-repository to promote access.

### Dissemination and future perspectives

This review will be published in peer-reviewed journals, and when possible, as a multipart series. We foresee that the scope of evidence derived from this review will allow for stratified reporting on the availability (phase 1) of PROMs, as well as the quality of PROMs (phase 2). Furthermore, when the scope of evidence allows it, we can consider stratified reporting based on specific domains of the ICF (e.g. activity, participation), disease profiles, or quality aspects (e.g. content validity). As referred to, the global burden of disease study, in conjunction with the ICF model, will be used as a framework to map the availability of PROMs (see phase 1) [13]. In addition to journal articles, results of this review will be (i) presented at global and continental scientific conferences, and (ii) a technical and executive summary will be drafted and shared with professional bodies and other stakeholders within the African continent that focus on clinical research.

The team foresees important future perspectives that further aim to disseminate and strengthen the review findings. Firstly, it is the ambition that the PROMs which are identified through this review will be aggregated in an online, open-access repository. The review of measurement properties will provide invaluable evidence to add to this repository that can assist stakeholders in the decision making, use, and appraisal of PROMs specific for their context. Similar platforms exist, for example for clinical assessment outcomes (i.e. PROQOLID™; eprovide.mapi-trust.org) or meetinstrumentenzorg.nl; yet these are not tailored to the unique African setting. The underlying belief is that this repository may facilitate wider access to the PROMs reviewed, and their evidence-base, for use in clinical practice or academic ventures. Direct access or reference to the instrument may be included, depending on the PROM’s licensing model; where needed we can work with or incentivize stakeholders to unlock the use of specific PROMS more easily and promote access. Once a repository has been established many other parameters of interest may be added, including detailed information on PROM analyses (e.g. scoring methods, cut-off values, handling of missing data, additional languages, other modes of administration, amongst others). The research team aims to closely work with a diverse set of stakeholders to help guide both content and ways of dissemination of the repository to cater for all potential users. Second, consensus-based (e.g. through Delphi methods) recommendations can be developed for prioritizing the translation, development, and/or validation of additional or new PROMs. These recommendations could, for instance, be informed by the local burden of disease (e.g. disease-specific gaps), specific populations being excluded from research (e.g. language- or culture-specific gaps), or perceived relevance for specific outcomes in driving health policy (e.g. outcome-specific gaps). Mapping the availability of PROMs against existing frameworks (i.e. burden of disease, ICF model) may provide a starting point for such a consensus procedure. Participatory action research methods can be used to engage patients and stakeholders in the prioritization process, to ensure that outcomes relevant to and valued by the patient are equally considered to those relevant for health policy. Finally, it is the ambition of the research team, through collaboration with stakeholders, that key PROMs will be recorded using voice-overs to ensure that visually impaired or illiterate individuals are not left behind.

### Limitations

It is apparent that with an average of 57 living languages per country, some of which may not be “just spoken” (e.g. hand signals) or some “just spoken” (e.g. not written), it is impossible to include all languages in this review. Besides, as indicated, there is a substantial proportion of the sub-Saharan African population that is illiterate. Hence, while this review is comprehensive, many languages and voices are still excluded. However, to retain its feasibility, we have opted to include those languages that are widely spoken (> 10 million people), supplemented by national languages where applicable. Once an online repository is established, one could add additional languages on a per case basis and potentially use more contemporary methods to include non-written languages. Furthermore, we acknowledge that while having culturally valid and language-appropriate PROMs may assist research specifically, this is merely one aspect within a wider set of transformative aspects that may need to be addressed to further promote inclusivity, including power dynamics, scientific trust, and cultural competencies [30]. A second limitation, given that > 169 million people in sub-Saharan Africa speak English, is that a similar type of review for PROMs validated in a sub-Saharan African setting in English may be valuable as well, in particular concerning the cross-cultural validity of English PROMs. Though, for reasons of pragmatism (i.e. scope of evidence) and feasibility, we chose to focus the present review specifically on those PROMs not in English. Thirdly, as the review and search strategy are focussed on the review of academic literature, there is a chance that PROMs that have been developed and studied in local languages may not find their way to the generally English peer-reviewed literature and will therefore be missed in the literature search. To partly address this limitation, three strategies will be implemented. First, a content-specific database (AfricaWide) is included in the search strategy. Second, the inclusion of grey literature through forward citation hashing, and thirdly, corresponding authors of articles included in the review will be contacted and asked to advise (and provide documentation where possible) on any unidentified PROMs in the languages stipulated.

## Conclusion

This protocol describes a comprehensive review for the identification of patient-reported outcome measures, and their measurement properties, that are developed, translated, and/or validated in non-English national or widely spoken languages in sub-Saharan Africa. The outcome of this review will provide an invaluable resource for clinicians and academics in the field and aims to promote inclusivity in health care and research.

## Supplementary Information

**Additional file 1.**

**Additional file 2.**

## Data Availability

Not applicable

## Abbreviations

COSMIN: COnsensus-based Standards for the selection of health Measurement INstruments
GRADE: Grading of Recommendations Assessment, Development, and Evaluation
ObSROM/s: Observer-reported outcome measure(s)
PROM/s: Patient-reported outcome measure(s)
WHO: World Health Organization
