# Supplementary material for: Patient-Reported OUtcome measures in key African languages to promote Diversity in research and clinical practice (PROUD)—protocol for a systematic review of measurement properties
Source: Trials. 2021 Jun 5;22:380. doi: 10.1186/s13063-021-05328-z (PMC8180136; doi:10.1186/s13063-021-05328-z)
Supplement: Supplementary file 2 — Additional file 2. [file 13063_2021_5328_MOESM2_ESM.docx]

# Example search strategy - PubMed

## Countries

#1. ((((((((((((((((((((((((((((((((((((((((((("angola"[MeSH Terms] OR "benin"[MeSH Terms]) OR "botswana"[MeSH Terms]) OR "Burkina Faso"[MeSH Terms]) OR "burundi"[MeSH Terms]) ) OR "cameroon"[MeSH Terms]) OR "Central African Republic"[MeSH Terms]) OR "chad"[MeSH Terms]) OR "comoros"[MeSH Terms]) OR "congo"[MeSH Terms]) OR "democratic republic of the congo"[MeSH Terms]) OR "Cote d'Ivoire"[MeSH Terms]) OR "djibouti"[MeSH Terms]) OR "eritrea"[MeSH Terms]) OR "ethiopia"[MeSH Terms]) OR "gabon"[MeSH Terms]) OR "gambia"[MeSH Terms]) OR "ghana"[MeSH Terms]) OR "kenya"[MeSH Terms]) OR "lesotho"[MeSH Terms]) OR "liberia"[MeSH Terms]) OR "madagascar"[MeSH Terms]) OR "malawi"[MeSH Terms]) OR "mali"[MeSH Terms]) OR "mauritius"[MeSH Terms]) OR "mauritania"[MeSH Terms]) OR "mozambique"[MeSH Terms]) OR "namibia"[MeSH Terms]) OR "niger"[MeSH Terms]) OR "nigeria"[MeSH Terms]) ) OR "rwanda"[MeSH Terms]) OR "senegal"[MeSH Terms]) OR "Sierra Leone"[MeSH Terms]) OR "somalia"[MeSH Terms]) OR "sudan"[MeSH Terms]) OR "South Africa"[MeSH Terms]) OR "eswatini"[MeSH Terms]) OR "tanzania"[MeSH Terms]) OR "togo"[MeSH Terms]) OR "uganda"[MeSH Terms]) OR "zambia"[MeSH Terms]) OR "zimbabwe"[MeSH Terms])

#2. "Angola" OR "Benin" OR "Botswana" OR "Burkina Faso" OR "Burkina Fasso" OR "Upper Volta" OR "Burundi" OR "Urundi" OR "Cabo Verde" OR "Cape Verde" OR "Cameroon" OR "Cameroons" OR "Cameron" OR "Central African Republic" OR "Chad" OR "Comoros" OR "Comoro Islands" OR "Comores" OR "Mayotte" OR "Congo" OR "Zaire" OR "Cote d'Ivoire" OR "Ivory Coast" OR "Djibouti" OR "French Somaliland" OR "Eritrea" OR "Ethiopia" OR "Gabon" OR "Gambia" OR "Ghana" OR "Gold Coast" OR "Kenya" OR "Lesotho" OR "Basutoland" OR "Liberia" OR "Madagascar" OR "Malawi" OR "Namibia" OR "Nyasaland" OR "Mali" OR "Mauritius" OR "Mauritania" OR "Mozambique" OR "Niger" OR "Nigeria" OR "Republic of Guinea" OR "Rwanda" OR "Sao Tome" OR "Senegal" OR "Sierra Leone" OR "Somalia" OR "South Africa" OR "Sudan" OR "Swaziland" OR "Tanzania" OR "Togo" OR "Togolese Republic" OR "Uganda" OR "Yemen" OR "Zambia" OR "Zimbabwe"

## Patient-reported Outcomes

#3. (((((((((((("HR-PRO"[Title/Abstract] OR "HRPRO"[Title/Abstract]) OR "HRQL"[Title/Abstract]) OR "HRQoL"[Title/Abstract]) OR "QL"[Title/Abstract]) OR "QoL"[Title/Abstract]) OR "quality of life"[Text Word]) OR "life quality"[Text Word]) OR "health index*"[Title/Abstract]) OR "health indices"[Title/Abstract]) OR "health profile*"[Title/Abstract]) OR "health status"[Text Word]) OR (((((("patient"[Title/Abstract] OR "self"[Title/Abstract]) OR "child"[Title/Abstract]) OR "parent"[Title/Abstract]) OR "carer"[Title/Abstract]) OR "proxy"[Title/Abstract]) AND ((((("report"[Title/Abstract] OR "reported"[Title/Abstract]) OR "reporting"[Title/Abstract]) OR (("rated"[Title/Abstract] OR "rating"[Title/Abstract]) OR "ratings"[Title/Abstract])) OR "based"[Title/Abstract]) OR (("assessed"[Title/Abstract] OR "assessment"[Title/Abstract]) OR "assessments"[Title/Abstract])))) OR ((((((((("disability"[Title/Abstract] OR "function"[Title/Abstract]) OR "functional"[Title/Abstract]) OR "functions"[Title/Abstract]) OR "subjective"[Title/Abstract]) OR "utility"[Title/Abstract]) OR "utilities"[Title/Abstract]) OR "wellbeing"[Title/Abstract]) OR "well being"[Title/Abstract]) AND (((((((((((((((("index"[Title/Abstract] OR "indices"[Title/Abstract]) OR "instrument"[Title/Abstract]) OR "instruments"[Title/Abstract]) OR "measure"[Title/Abstract]) OR "measures"[Title/Abstract]) OR "questionnaire"[Title/Abstract]) OR "questionnaires"[Title/Abstract]) OR "profile"[Title/Abstract]) OR "profiles"[Title/Abstract]) OR "scale"[Title/Abstract]) OR "scales"[Title/Abstract]) OR "score"[Title/Abstract]) OR "scores"[Title/Abstract]) OR "status"[Title/Abstract]) OR "survey"[Title/Abstract]) OR "surveys"[Title/Abstract]))

## Language

#4. Swahili OR French OR Arabic OR Hausa OR Pidgin OR Amharic OR Yoruba OR Portuguese OR Igbo OR Zulu OR Sotho OR Xhosa OR Oromo OR Afrikaans OR Fulfulde OR Somali OR Setswana OR Wolof OR Jula OR Kinyarwanda OR Rundi OR Ibibio OR Malagasy OR Tsonga OR Sango OR Swati OR Ndebele OR Venda OR Creole OR Spanish OR Kabuverdianu

**Final search phase 1**

(#1 OR #2) AND #3 AND #4

## Measurement properties

#5. ((instrumentation[sh] OR "Validation Studies"[pt] OR "reproducibility of results"[MeSH Terms] OR reproducib*[tiab] OR "psychometrics"[MeSH] OR psychometr*[tiab] OR clinimetr*[tiab] OR clinometr*[tiab] OR "observer variation"[MeSH] OR "observer variation"[tiab] OR "discriminant analysis"[MeSH] OR reliab*[tiab] OR valid*[tiab] OR coefficient[tiab] OR "internal consistency"[tiab] OR (cronbach*[tiab] AND (alpha[tiab] OR alphas[tiab])) OR "item correlation"[tiab] OR "item correlations"[tiab] OR "item selection"[tiab] OR "item selections"[tiab] OR "item reduction"[tiab] OR "item reductions"[tiab] OR agreement[tiab] OR precision[tiab] OR imprecision[tiab] OR "precise values"[tiab] OR test-retest[tiab] OR (test[tiab] AND retest[tiab]) OR (reliab*[tiab] AND (test[tiab] OR retest[tiab])) OR stability[tiab] OR interrater[tiab] OR inter-rater[tiab] OR intrarater[tiab] OR intra-rater[tiab] OR intertester[tiab] OR inter-tester[tiab] OR intratester[tiab] OR intra-tester[tiab] OR interobserver[tiab] OR inter-observer[tiab] OR intraobserver[tiab] OR intra-observer[tiab] OR intertechnician[tiab] OR inter-technician[tiab] OR intratechnician[tiab] OR intra-technician[tiab] OR interexaminer[tiab] OR inter-examiner[tiab] OR intraexaminer[tiab] OR intra-examiner[tiab] OR interassay[tiab] OR inter-assay[tiab] OR intraassay[tiab] OR intra-assay[tiab] OR interindividual[tiab] OR inter-individual[tiab] OR intraindividual[tiab] OR intra-individual[tiab] OR interparticipant[tiab] OR inter-participant[tiab] OR intraparticipant[tiab] OR intra-participant[tiab] OR kappa[tiab] OR kappa's[tiab] OR kappas[tiab] OR repeatab*[tiab] OR ((replicab*[tiab] OR repeated[tiab]) AND (measure[tiab] OR measures[tiab] OR findings[tiab] OR result[tiab] OR results[tiab] OR test[tiab] OR tests[tiab])) OR generaliza*[tiab] OR generalisa*[tiab] OR concordance[tiab] OR (intraclass[tiab] AND correlation*[tiab]) OR discriminative[tiab] OR "known group"[tiab] OR "factor analysis"[tiab] OR "factor analyses"[tiab] OR dimensionality[tiab] OR subscale*[tiab] OR "multitrait scaling analysis"[tiab] OR "multitrait scaling analyses"[tiab] OR "item discriminant"[tiab]OR "interscale correlation"[tiab] OR "interscale correlations"[tiab] OR ((error[tiab] OR errors[tiab]) AND (measure*[tiab] OR correlat*[tiab] OR evaluat*[tiab] OR accuracy[tiab] OR accurate[tiab] OR precision[tiab] OR mean[tiab])) OR "individual variability"[tiab] OR "variability analysis"[tiab] OR (uncertainty[tiab] AND (measurement[tiab] OR measuring[tiab])) OR "standard error of measurement"[tiab] OR sensitiv*[tiab] OR responsive*[tiab] OR (small*[tiab] AND (real[tiab] OR detectable[tiab]) AND (change[tiab] OR difference[tiab])) OR "meaningful change"[tiab] OR "minimal important change"[tiab] OR "minimal important difference"[tiab] OR "minimally important change"[tiab] OR "minimally important difference"[tiab] OR "minimal detectable change"[tiab] OR "minimal detectable difference"[tiab] OR "minimally detectable change"[tiab] OR "minimally detectable difference"[tiab] OR "minimal real change"[tiab] OR "minimal real difference"[tiab] OR "minimally real change"[tiab] OR "minimally real difference"[tiab] OR "ceiling effect"[tiab] OR "floor effect"[tiab] OR "item response model"[tiab] OR irt[tiab] OR rasch[tiab] OR "differential item functioning"[tiab] OR dif[tiab] OR "computer adaptive testing"[tiab] OR "item bank"[tiab] OR "cross-cultural equivalence"[tiab]) NOT ("addresses"[Publication Type] OR "biography"[Publication Type] OR "case reports"[Publication Type] OR "comment"[Publication Type] OR "directory"[Publication Type] OR "editorial"[Publication Type] OR "festschrift"[Publication Type] OR "interview"[Publication Type] OR "lectures"[Publication Type] OR "legal cases"[Publication Type] OR "legislation"[Publication Type] OR "letter"[Publication Type] OR "news"[Publication Type] OR "newspaper article"[Publication Type] OR "patient education handout"[Publication Type] OR "popular works"[Publication Type] OR "congresses"[Publication Type] OR "consensus development conference"[Publication Type] OR "consensus development conference, nih"[Publication Type] OR "practice guideline"[Publication Type]) NOT ("animals"[MeSH Terms] NOT "humans"[MeSH Terms]))

**Final search phase 2**

(#1 OR #2) AND [SPECIFIC PROM] AND #4 AND #5
